# The next frontier of the anaerobic digestion microbiome: From ecology to process control

**DOI:** 10.1016/j.ese.2020.100032

**Published:** 2020-05-08

**Authors:** Jo De Vrieze

**Affiliations:** Center for Microbial Ecology and Technology (CMET), Ghent University, Coupure Links 653, B-9000, Gent, Belgium

**Keywords:** Amplicon sequencing, Anaerobic digestion, Fingerprinting, Methanogenesis, Microbiome

## Abstract

The anaerobic digestion process has been one of the key processes for renewable energy recovery from organic waste streams for over a century. The anaerobic digestion microbiome is, through the continuous development of novel techniques, evolving from a black box to a well-defined consortium, but we are not there yet. In this perspective, I provide my view on the current status and challenges of the anaerobic digestion microbiome, as well as the opportunities and solutions to exploit it. I consider identification and fingerprinting of the anaerobic digestion microbiome as complementary tools to monitor the anaerobic digestion microbiome. However, data availability, method-inherent biases and correct taxa identification hamper the accuracy and reproducibility of anaerobic digestion microbiome characterization. Standardisation of microbiome research in anaerobic digestion and other engineered systems will be essential in the coming decades, for which I proposed some targeted solutions. These will bring anaerobic digestion from a single-purpose energy-recovery technology to a versatile process for integrated resource recovery. It is my opinion that the exploitation of the microbiome will be a driver of innovation, and that it has a key role to play in the bio-based economy of the decades to come.

## Highlights

• Identification and fingerprinting characterize the anaerobic digestion microbiome.
• The anaerobic digestion microbiome reflects multiple challenges and opportunities.
• The exploitation of the anaerobic digestion microbiome is a driver of innovation.

## 1 Introduction

The recovery and supply of renewable energy can be considered one of the key challenges of our present changing World with its increasing human population. The European Commission recently (end 2019) engaged an ambitious commitment to achieve no net greenhouse gas emission by 2050. This so-called European Green Deal (COM/2019/640 final), obviously implies that other international partners share this ambition to avoid “carbon leakage” outside the European Union [1].

Multiple renewable energy supply systems have been put forward to contribute to this ambitious objective, including photovoltaics, on- and offshore wind, hydropower, geothermal, and biomass [2,3]. One of the key technologies for renewable energy recovery from biomass is anaerobic digestion, which allows the production of energy-rich biogas. Renewable energy represented about 18% of total gross final energy consumption in 2018 in the EU [4]. Biogas constitutes about 8% of the renewable energy supply (estimation 2015), with values still increasing each year [5]. These numbers indicate that anaerobic digestion is not the major solution for renewable energy recovery, yet, here, I will emphasize the vast potential of this technology, through the exploitation of the anaerobic digestion microbiome, and the possibilities for its future development beyond mere energy recovery. Hence, the key objective of this perspective is to provide solutions to the existing and emerging issues concerning microbiome engineering in anaerobic digestion, and consider future research directions for the coming decades.

## 2 The anaerobic digestion microbiome

### 2.1 Status and challenges

Anaerobic digestion is a process that relies on a well-balanced mixed microbial community, with a history that dates back almost 140 years [6]. Over these years, knowledge concerning the key process parameters and microbial community composition, organisation and activity has increased alongside the development of novel monitoring techniques and molecular/microbial methods. The identification of key process parameters, *i.e.*, total ammonia (NH_4_^+^ + NH_3_), residual volatile fatty acids (VFA), pH, salinity, temperature, and sludge retention time [7,8], has been rather straightforward. Recently more advanced and/or integrated physico-chemical monitoring approaches have been developed, either for early-warning indication [9,10] and/or based on alternative parameters or through novel methods [[11], [12], [13]].

Monitoring and/or evaluation of the microbial community can be considered on two levels, *i.e.*, the identification of taxa, genes, transcripts, proteins and metabolites or the characterization of the microbial community as a coherent entity through fingerprinting, each of which, in my opinion, has its merits. For argument sake, here, I will consider this approach for taxa, but this applies to genes, transcripts and metabolites as well, all of which, in my opinion, will become more and more prominent for microbiome-based process engineering in the decades to come. The first level concerns the identification of taxa at different phylogenetic levels, which enabled the identification of a “core microbiome” [[14], [15], [16], [17]], and indicator taxa of process performance [[18], [19], [20]]. Moreover, each individual anaerobic digestion was confirmed to host a unique microbiome [21,22]. While the identification of taxa enables monitoring of key taxa with early-warning potential for process failure detection [23], it does not consider the overall concept of redundancy [24], which can be considered of key importance in anaerobic digestion [[25], [26], [27]]. Such monitoring of dynamics, related to redundancy, requires another approach that provides an overall view on the microbial community through fingerprinting.

Microbial fingerprinting can be considered the second level of microbial community monitoring, and does not necessarily imply the need for taxa, genes, transcripts, proteins or metabolites identification. This enables the usage of more traditional methods, such as terminal restriction length polymorphism (T-RFLP) [28,29], or denaturing gradient gel electrophoresis [30,31]. Even though these “old-school” techniques have their merits, high throughput amplicon sequencing and the “omics” should be the preferred method**s**, because they allow both identification and overall microbial community fingerprinting. Fingerprinting approaches will, in the decades to come, exceed the level of DNA-based amplicon sequencing to RNA-based approaches, either at the 16S level [32] or the entire transcriptome, and to phenotypic fingerprinting through flow cytometry [33,34]. Such fingerprints capture both α-diversity, *e.g.*, richness, evenness and overall diversity [35], and β-diversity parameters to characterize both (dis)similarity and dynamics in and between anaerobic digesters [18]. Even though these fingerprinting techniques estimate microbial community organisation (α-diversity) and the key stress response mechanism of the microbial community, *i.e.*, resistance, resilience or redundancy [24,36], they fail to identify key taxa, genes, transcripts, proteins and metabolites, and commonly do not provide a direct answer concerning microbial community performance [37]. Hence, a combined complementary approach of key taxa, genes, transcripts or metabolites identification and overall microbial community fingerprinting at different levels is, in my opinion, essential for the transition to a new level of “microbial community monitoring” in anaerobic digestion.

**Fig. 1:**
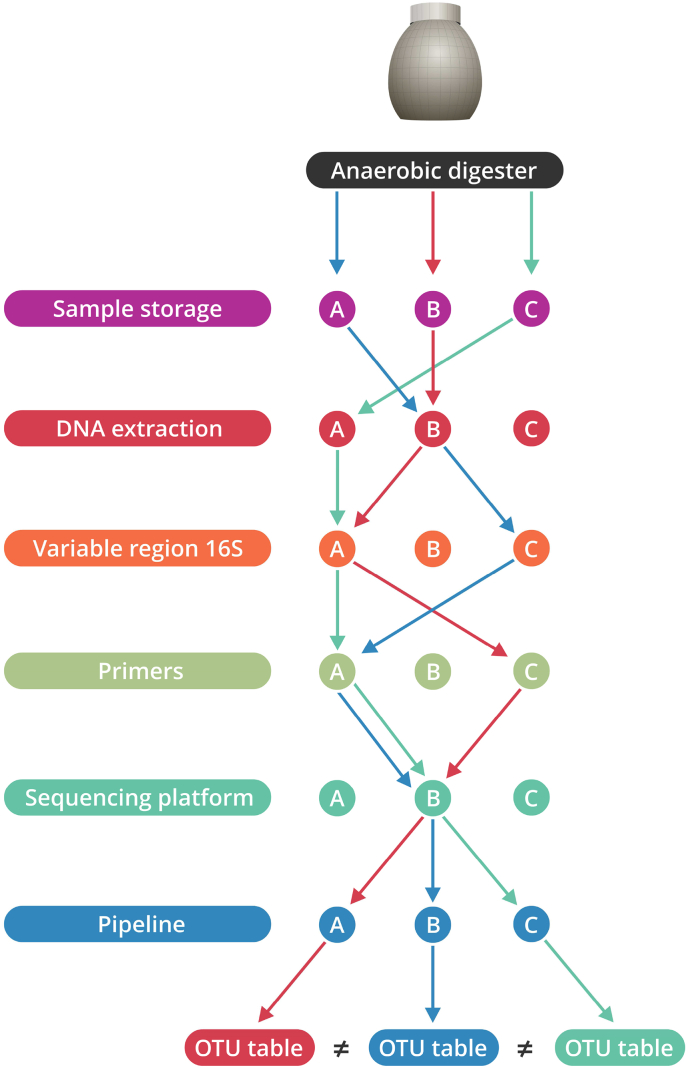
Schematic overview of the potential method-inherent biases in the process of obtaining a microbial community profile (operational taxonomic unit (OTU) table) from an anaerobic digester. For each step in the process, three potential options are listed (even though more are possible), resulting in a total of 3^6^ possibilities and potentially different microbial community profiles. The differently coloured arrows (blue, green and red) reflect a different sequence of method selection, resulting in a potentially different microbial community profile. (For interpretation of the references to colour in this figure legend, the reader is referred to the Web version of this article.)

Microbial community monitoring in anaerobic digestion, but also other microbial ecosystems, comes at the cost of method-inherent biases and the lack of, even research field-specific, standardisation [38]. The succession of sample storage, DNA extraction method, primer choice, variable region in the 16S rRNA gene selection, amplicon sequencing platform and data processing pipeline entails a series of choices that can strongly impact the final microbial community profile (Fig. 1). If the same methods are used, reliable and robust results can be obtained, yet, this complicates data comparison between studies using different methods. Already at the sample storage, microbial community composition and fingerprints can be influenced, as demonstrated for faecal samples [39]. The subsequent DNA extraction of samples from anaerobic digesters appears to have a protocol-related effect on the microbial community profile [40], which can even be influenced by minor modifications in the protocol [41]. The primer choice, whether or not in relation to a difference in the variable or V-region in the 16S rRNA gene, can also strongly impact the microbial community profile [[41], [42], [43]]. Even though Illumina has become the standard for amplicon sequencing, amongst others with the discontinuation of 454 pyrosequencing in 2013, the Ion Torrent, Pacific Biosciences, and especially the Nanopore MinION technology [44] are becoming more comment. However, their different microbial community profile outcomes, which was observed even for mock microbial communities [43] and genomic analyses [45] will also have its reflection on the anaerobic digestion microbial community profile. Finally, the data processing pipeline (*e.g.*, Mother, QIIME 1&2), and its (inherent) settings have been shown to influence mock community profiles [46], which undoubtedly also reflects the profile of the complex microbial community in anaerobic digestion.

These inherent method-related biases constitute an immense and expanding challenge in anaerobic digestion, but also other (engineered) microbial ecosystems. A key question remains whether one is looking at the actual microbial community or rather at method-related artefacts, which, in turn, makes one question the entire concept of microbial community amplicon sequencing in anaerobic digestion (and beyond). Fingerprinting appears to be less method-dependent than taxon identification, as observed when comparing T-RFLP with Illumina amplicon profiles [47,48], but, as mentioned earlier, should be combined with taxon identification. Hence, this constitutes a, to my opinion, key challenge in future research and method standardisation to enable accurate and reliable microbial community monitoring in anaerobic digestion.

### 2.2 Opportunities and solutions

Fifteen years of high-throughput amplicon sequencing [49] have resulted in a vast amount of studies concerning the anaerobic digestion microbiome, either in lab-scale experiments or full-scale digesters. Several researchers carried out meta data studies [50,51], each of which expanded our overall knowledge on the anaerobic digestion microbiome, but also stumbled upon several issues/opportunities. A first issue concerns the availability of both raw (fastq) and processed (operational taxonomic unit (OTU) table) data files. Generally, these data files should be made accessible through online depositories, *e.g.*, the National Center for Biotechnology Information (NCBI) or the European Nucleotide Archive (ENA). However, especially in the early era of high-throughput sequencing, authors often waived such responsibility, due to various, often valid, reasons. This makes it impractical for other researchers to access these valuable data. It is my view that both raw (online repositories) and processed (in supplementary information or online repositories) data should be provided directly to the reader to allow them to (1) directly use the raw files and (2) compare their outcome following data processing with the processed data of the author.

The second issue emerges from the first one, *i.e.*, the diverse use of primer sets, V-regions and sequencing platforms makes comparison of raw data sets a challenging task. To tackle this issue, three essential actions are needed. First, a comprehensive study that documents the impact on the anaerobic digestion microbiome (both taxon identification and fingerprinting) of DNA extraction method, primer choice, variable region in the 16S rRNA gene selection, amplicon sequencing platform and data processing pipeline will be critical. This should be done by merging existing studies on lab- and full-scale. Second, this information should enable selecting (a set of) suitable techniques that act as standard to avoid or at least limit future method-based biases in amplicon sequencing techniques. A similar method standardisation approach was applied for the earth microbiome project [52] and human microbiome project [53], which should also find its way into anaerobic digestion and other engineered microbial ecosystems. Third, following the selection of standardised method, the key parameters to be reported in relation to anaerobic digestion microbiome data, especially at the full-scale, should be selected, as, currently, operational data inclusion is quite variable between studies.

A third issue concerns the identification of key taxa, which, even when method-related biases are eliminated, poses an important challenge with respect to the ever-evolving, but still incomplete multitude of microbial databases [54]. Ecosystem-specific databases, for example, the MiDAS database for activated sludge [55] and anaerobic digestion [56], could provide an answer to this problem, through a more target-oriented identification approach of microbial taxa. Nonetheless, constant updating, which is meticulously done for the MiDAS database [57], is essential to keep the ecosystem-specific databases in line with the global databases. Full-length 16S rRNA gene sequencing makes it even possible to establish a more complete ecosystem-targeted database, for the activated sludge and anaerobic digestion in the case of MiDAS, exceeding the limitations of large-scale general databases [57]. The application of amplicon sequence variants (ASVs) instead of OTUs, which allows finer resolution, independently from reference databases, to single-nucleotide differences [58], is an approach that could allow more accurate identification of taxa. The identification and characterization of novel taxa, to be incorporated in microbial databases, remains a key issue for anaerobic digestion, given the (1) anaerobic conditions and (2) complex interactions, which makes pure culture growth of key taxa challenging. Even though we are in an era of high-throughput methods that (partially) replace classic microbiology approaches, as is the case in for example drinking water [59], (basic) microbiology will remain important in the decades to come to solidify and expand or knowledge on the anaerobic digestion microbiome.

I illustrated these challenges on the level of amplicon sequencing, as these have become imminent in the previous decade, yet, these challenges equally apply to the “omics”, which, in my opinion, will become apparent in the coming decades. The key challenge is to overcome these issues, which I prefer to consider as challenges/opportunities, in the coming decades, and we have the techniques/knowledge to do so. This will allow us to expand our knowledge of the anaerobic digestion microbiome, with the potential to shift from mere post hoc description to integrated and proactive process steering and engineering.

## 3 The future of the anaerobic digestion microbiome

As Voltaire so eloquently phrased in the 18th century: “Le mieux est l’ennemi du bien. (Better is the enemy of good.)”. The vision of using microbiome-based parameters to monitor and steer the anaerobic digestion process should expand beyond the border of current knowledge, and should bring forth novel opportunities, rather than rephrasing or confirming current process steering approaches. Anaerobic digestion as a process has gone through more than a century of development and optimization [6], but currently reaches its limits, related to the inherently low economic value of biogas. The pressing issues of Climate Change, translated in the European Green Deal, will require anaerobic digestion to move towards a new level to keep up with other renewable energy providing technologies, such as photovoltaics and wind energy. Hence, anaerobic digestion needs to evolve as a process beyond mere energy recovery to integrated resource recovery, or will perish alongside other technologies that are unable to deal with today’s sustainability requirements.

The anaerobic digestion microbiome can play a key role in this transition, if we succeed in successfully completing the above-mentioned opportunities. The anaerobic digestion microbiome knowledge can provide us with key/indicator/marker (whatever you prefer to name them) taxa to monitor, for which we can develop suitable methods. It will allow us to track microbiome stability through fingerprinting, for which we should develop suitable, either case-specific or general, benchmarks. It will enable us to exceed beyond taxa to key/indicator/marker genes (and their transcripts) and pathways (through application of the “omics”) that will predict the potential of the anaerobic digestion process, be it feedstock degradation potential, organic loading capacity or stress tolerance. A key example of such an approach with high potential resides in the monitoring of the F_420_ cofactor through flow cytometry [34] or the F_430_ cofactor through liquid chromatography [60], both of which can provide an accurate and direct view on methanogenic activity. It will allow us to not only monitor taxa, but also apply strategies to selectively enrich taxa (and even genes and pathways) beyond the limited potential of bioaugmentation [61].

These opportunities do not conflict with the initial citation of Voltaire, as further development of the anaerobic digestion process is essential for it to cope with today’s (emerging) challenges. Anaerobic digestion has the potential to expand its central role in the bio-based circular economy, as a beacon of not only renewable energy recovery, but also valorisation of novel/challenging waste streams, *e.g.*, within the biorefinery, integrated resource recovery (N, P, K and beyond) and targeted product outcome beyond biogas towards commodity chemicals, *e.g.*, through super-dry reforming [62], polyhydroxyalkanoates, medium- and long-chain fatty acids [63], and microbial protein for food/feed applications [64]. This will re-invent anaerobic digestion as a key process in the bio-based circular economy in which microbial ecology will, thus, play a key role. Hence, it is my vision that the anaerobic digestion microbiome will be the driver of innovation of the anaerobic digestion process as such and mixed culture fermentation processes in general to tackle the main challenges of our present society in the decades to come.

## Declaration of competing interest

The authors declare that they have no known competing financial interests or personal relationships that could have appeared to influence the work reported in this paper.
